# 

**Published:** 1850-10-01

**Authors:** 


### BETHLEM HOSPITAL.—MEDICAL SCHOOL.

THE Winter Term for the Study of Mental Diseases in Bethlem Hospital will commence on Tuesday, the 15th of October next, and terminate on Saturday, the 15th of March, 1851.

Members of the Medical Profession and Medical Students, desirous of attending the Hospital Practice, are required to enter their names at the Hospital on or before Saturday, the 12th of October next. Admission Fee, 3*l.* 3*s.*

The Physicians, and in their absence the Apothecary, will enter Students, and give all necessary information.

*Sept.* 18, 1850. B. WELTON, Clerk.

### APPOINTMENTS.

Dr. Davey, late of Hanwell and Ceylon, and Dr. Hood, have been selected by the Visiting Middlesex Magistrates to fill the posts of resident medical superintendents of the Colney Hatch Lunatic Asylum, Middlesex.

### To Correspondents.

As our correspondence, in connexion with the Journal, has become rather extensive—stretching to the remote parts of India, France, Germany, and America—we must crave the indulgence of our kind friends. In our next, we propose publishing No. 2 of the Editor's Portfolio. We again solicit from our subscribers, at home and at a distance, the latest reports of lunatic asylums, and any psychological information that may interest our readers. Several American periodicals have reached us, which we have reluctantly been compelled to refuse, in consequence of the enormous postage charged. For a "Charleston Journal," the postage demanded was one pound! All these periodicals might be sent, with little expense, through respectable publishers.

### INDEX TO VOL. III.

Abendberg institution for cretinism, 58
Admissions into lunatic asylums, the forms prescribed a sufficient protection to the public, 437
Age, its effects on the mind, 213
Agriculture useful occupation for the insane, 482
Amentia, progressive steps to, from reason, by Pinel, 157
American reports of lunatic asylums. *Vide* Reports.
Ancients, their knowledge and treatment of insanity, 171
———, their psychological speculations, 503
Annales Medico-Psychologiques, selections from, 218
Anti-materialism *versus* materialism, 272
Asclepiades, mode of treating insanity, 171
Asylum reports. *Vide* Reports.
Attendants in asylums, qualifications necessary for, 347, 353
Autobiography of the insane, 40
Baillarger, Dr., influence of political revolutions on the number of the insane, 36
———, on erysipelas of the face and scalp with general paralysis, 218
Bathing, warm and cold, recommended, 181
Baths, prolonged warm water, rules for their administration, by Brierre de Boismont, 182
Bayle, M., on general paralysis, 240
Bedford Lunatic Asylum Report, 86
Belfast Lunatic Asylum Report, 86
Belhomme, Dr., on the causes of insanity from popular commotions, 31
Belladonna, authorities in favour of its administration, 176
Bentley's Miscellany and mad doctors, 123
Bethlem, history of its foundation, 429
Billing, Dr., his first principles of medicine, 138
Bingham, Dr., classification of mental disorders. 275
Bird, Dr. Golding, on electricity and galvanism, 189
Bleeding, evidence for and against its practice in insanity, 179
———, observations on, 483
Bondi, case of periodical mania, 132
Boismont, Brierre de, directions for prolonged bathing, 182
———, on tædium vitæ, 545
Brain, human, its anatomy, 197
———, physiology, 373
———, its fœtal development, 197
Brain, a double organ, 11
———, morbid appearance in epileptics, 365
———, morbid appearances in mania, 235
———, its ramollissement, 244
———, its connexion with the mind, 383
———, its pathology in insanity, 228, 362, 501
Browne, Dr., on the melancholia, sleeplessness, insensibility, and discontent of the insane, 89, 440
Calmeil, on the pathology of epilepsy, 240
Camphor, authorities in favour of its administration, 17
Cerebellum, its functions, 377
Ceylon, state of the insane, reported by Dr. Davey, 326
Chambers, Dr., on corpulency, 566
Charlesworth, Dr., first who abolished every form of restraint, 441
Chloroform, effects of its administration, by Dr. M'Gavin, 93
———, its bad effects during parturition, by Dr. Webster, 269
Classification of mental disorders, 275
———, tables of, 486
Colney Hatch Asylum, on the appointment of its medical staff, 384
Commissioners in lunacy, information required by them on the different modes of medical treatment adopted in lunatic asylums, 178
———, their care that the forms of admission and medical certificates be correct, 437
———, their testimony of the improvement of private lunatic asylums, 439
———, their desire that all forms of restraint should be abolished, 441
Conolly, Dr., testimonial to, 565
Confessions of a lunatic lady, 388
Consumption, pulmonary, arrested by epilepsy, 368
Coote, Holmes, on the anatomy of the brain, 197
———, pathology of its membranes, 521
Copland, Dr. James, on palsy and apoplexy, 422
Corfe, Dr. G., on the physiognomy of disease, 137
Correspondence from Paris, 126
Cretinism, account of, in Switzerland, 54
Crichton institution, Dumfries Report, 67
Crime, analysis of, by R. H. Horne, 96
Criminals, victims of disease, 13

Danger to be apprehended in all cases of mental aberration, 17
Davey, Dr., on mental pathology, 334
Day, on mental diseases in advanced life, 212
——, his appointment to the Chandos professorship at St. Andrew's, 218
Deaths in prisons and asylums, 230
———, causes of, in epilepsy, 371
——— of females at Hanwell, from 1846 to 1850, with their respective ages, 504
Debate, amusing, on the use of tobacco by insane patients, 487
Delusions, religious, observations on, 474
Depravity, causes of moral, 184
Descuret, M., on the seat of the passions, 143
Digitalis, authorities in favour of its administration, 176
Donizetti, the composer, interesting anecdote of, 499
Dowler, on the natural history of death, 423
Downing, Dr. Toogood, on tic douloureux, 137
Dudley on anti-materialism, 273
Education, deficient, gives rise to want of self-control, 452
Epilepsy, epidemic, 10
———, its pathology not well understood, 239
———, pathological appearances in cases of, 364
———, the causes of death from, 371
Erotomania, its nature, 388
Errors, popular, respecting insanity, 347
Esquirol, his distinction between idiocy and dementia, 305
———, on hereditary insanity, 493
———, his reason for recommending seclusion, 177
Etat Mixte, a form of insanity, 492
Evening Thoughts, by a physician, 271
Expenses, annual, of lunatic asylums in America and Great Britain, 489
Falret, M., on the clinical study of mental disease, 225
Farming useful occupation for the insane, 482
Fascination a cause of crime, 277
Food, refusal of, Dr. Verga's plan of treatment, 219
Forbes's, Dr., Physician's Holiday, or a Month in Switzerland, 54
Foville, his opinion on general and topical bleeding, 179
France, the laws of lunacy in, 400
———, Dr. Webster's account of its asylums, and treatment of the insane, 529
Ganglia of the cerebro-spinal system described, 205
Gardening a useful occupation for the insane, 482
Gaskell, Samuel, Esq., congratulatory address to, 139
Goitre, its connexion with cretinism, 59
Grimes, Stanley, on mesmerism and magic, 422
Guggenbüll's establishment for cretins, 54
Hæmorrhage a cause of insanity, 175
Harrison, J. B., on the human mind in some of its medical aspects, 246
Heart, diseases of, complicated with paralysis, 245
Hereditary causes of idiocy, 318
——— predisposition to insanity, 91
——— transmission of the intellectual faculties and propensities, 492, 499
Hitchman's lectures on the pathology of insanity, 228, 362, 501
———, his appointment to the Derby County Asylum, 424
Home treatment, its disadvantages, 342
Homicidal insanity, curious cases of, 49, 51, 465
Hopkins's, Dr. R. C., Reports of the Ohio State Lunatic Asylum, 456
Horne, R. H., analysis of crime, 94
Hospitals for the incurably insane, 354
——— for the insane, their expenses in America and Great Britain, 490
——— for the intemperate proposed, 358
Howe, Dr., on the causes and prevention of idiocy, 296
Hugman, W. C., on morbus coxarius, 274
Hyoscyamus used by the ancients in the treatment of insanity, 173
———, authorities in favour of its administration, 176
Hypochondriasis, observations on, 1
Hysteria complicated with amenorrhea, 226
Ice a therapeutic agent in cases of insanity, 128
Ideology, observations on, 193
Idiocy—its causes, cure, and modes of prevention, 292
Idiots, their number in Massachusetts, 296
—— their general physiological and physical condition, 306
—— their phrenological development, 309
Insane, autobiography of the, 40
Insanity defined, 16
———, its treatment by the ancients and moderns, 164
———, its form in advanced life, 213
———, peculiar form of mixed insanity, in which it is blended and co-exists with reason, 491
———, produced by chloroform, 269
———, causes of moral, 35
———, cases of, during the late French Revolution, 31
———, the existence of moral insanity, 258
———, popular errors respecting it, 347
———, lectures on its pathology, 228, 362, 501

Insanity, the verdict of a jury necessary to establish a case at Ohio, 461
———, sudden recovery from, to reason, 481
———, its peculiar type hereditary, 493
———, latent, observed in the visitors to asylums, 497
———, the power of self-control in resisting its invasion, 452
Instruction, its relation to crime, 103
Intellectual faculties, their decay in advanced life, 213
———, their abnormal activity in the mixed form of insanity, 495
Intemperance a cause of idiocy, 317
——— a phase of insanity, 357
Intoxication produced by iodide of potassium, 227
Italy slow in the improvement of its lunatic asylums, 435
Kent County Lunatic Asylum Report, 77
Kirkbride, Reports on the Pennsylvania Hospital for the Insane, 339
Kirkes, Dr. W. S., on the physiology of the brain, 373
Laws—the French law of lunacy, 400
Lectures on the pathology of insanity, by Dr. Hitchman, 228, 362, 501
Letter-writing a source of occupation and amusement to the insane, 85
Letters from recovered patients, giving an account of their malady, 472
Lunar influence on insanity, 485
Lunacy, observations on the English law, 14
———, the French law of, 400
Lunatics formerly treated with severity and cruelty in England—several hanged 430
———, their condition in Ceylon, 323
———, amelioration of their condition, and present comforts, 439
———, advantages of their associating together—sometimes cure one another, 443
———, capable of combining and conspiring together, 480
Lunatic asylums, public and private, their history and present state, 425
———, their general improvement during the last few years, 430. *Vide* Reports.
Lycanthropy, 10, 167
Mad doctors, absurdity of the designation, 123
Madness and reason combined, 491
Malcolm, Dr., on hereditary predisposition to insanity, 92
Mania, case of periodical, by M. Bondi, 132
Massachusetts Reports to the House of Representatives, on the condition of idiots in America, 293
Mc Gavin, Dr., successful administration of chloroform, 93
Meugy, history of an extraordinary nervous disorder, 221
Mind, considered in its medical aspects, 246
Moon, its influence on the insane, 485
Monomania, homicidal, remarkable cases of, 49, 51
Moore, Dr. George, health, disease, and remedy, 420
Moral insanity, 258
Moreau, Dr., on mixed insanity, 490
Morning Chronicle, its valuable articles on "Labour and the Poor," 183
Mortality in lunatic asylums and in prisons compared, 230
Morton, Mr., his lamented death, 19
Musk, authorities in favour of its administration, 176
Narcotics used in the treatment of insanity, 176
Nerves of the spinal cord and brain, 204
Nottidge *versus* Ripley, observations on the charge of Baron Pollock, 14
Occupation of the insane, its importance, 83
Ogilvie, Dr., lectures on insanity at Marischal College, Aberdeen, 557
Ohio lunatic asylum, 456
——— description of the building, grounds, and rules of government, 458
——— the prescribed forms of admission into, 463
——— interesting case of homicidal insanity at, 466
Oken, his theory of the skull, being a repetition of the vertebræ, 200
Opium, authorities in favour of its administration, 176
Orations of American independence, 475
Oration delivered in the Ohio asylum, 475
Padded rooms, invented by Dr. Autenreith, 441
Paralysis, general, its pathology, 242
——— complicated with diseases of the heart, 245
Passions, their influence on the body, 142
Pate, his trial, 557
——— just decision of the jury, 451
Pathology, mental, by Dr. Moreau, 490
Pennsylvania Hospital for the Insane, 340
Phrenology, how far useful in cases of insanity, 323
Phrenological development of idiots, 309
Pinel on the moral causes of insanity, 35
Politics and insanity, 31
Poverty, a cause of idiocy, 293
Psychologists, their authority in cases of disputed insanity vindicated, 447
Pulmonary diseases complicated with insanity, 233
Punishments, capital, psychologically considered, 61
Pyromania, remarkable case of, 419
Ramollissement of the brain, 244

Reason, sudden restoration to, 481
——— and madness, a form of mixed insanity, 490
Religion, editorial remarks in reply to a pamphlet on its relation with diseased mind, 283
——— in asylums, 468
Religious insanity, 283
Remedies, those chiefly recommended in the treatment of insanity, 176
Reports of British Lunatic Asylums—
Bedford Lunatic Asylum, 86
Belfast District Asylum, 86
Crichton Institution, Dumfries, 87
Dundee Royal Lunatic Asylum, 84
Forston County Lunatic Asylum, Dorset, 81
Friends' Retreat, near York, 76
Kent County Lunatic Asylum, 77
Montrose Lunatic Asylum, 92
Murray Royal Lunatic Asylum, 90
Suffolk Lunatic Asylum, 85
West Riding Lunatic Asylum, 93
Yorkshire Lunatic Asylum for the North and East Ridings, 77
Reports from America:—
To the Legislature of Massachusetts by the Commissioners appointed to inquire into the condition of idiots within the Commonwealth, first and second report, 293
The Ohio Lunatic Asylum, 456
The Pennsylvania Hospital for the Insane, 339
Restraint first abolished by Dr. Charlesworth at the Lincoln Asylum, 441
———, the question of restraint and non-restraint considered, 351
———, cases in which it was considered necessary, 77
———, excess of, in the French asylums, 539
Revolutions, a political cause of insanity, 35
Rewards and punishments, influence of, upon the insane, 454
Rodriguez on the pathology of epilepsy, 240
Rush, Dr., on lunar influence upon the insane, 486
Schools for idiots established in France, 295
Seasons, their influence upon insanity, 485
Seclusion of the insane necessary for their successful treatment, 177
Self-control, importance of, in the incipient stage of mental disease, 453
Smee, Alfred, on instinct and reason, 423
Smith, Sydney, elementary sketches of moral philosophy, 421
Sombre, Mr. Dyce, his complaints against the administration of the English law of lunacy, 410
Society, its moral state psychologically considered, 184
Somerset County Lunatic Asylum, 271
Spinal cord and brain, the deposition of nervous matter in, 197
Statistical tables on insanity, 486
Stone's, Dr., observations on the appointment of the medical staff at Colney Hatch Asylum, 384
Stramonium, authorities in favour of its administration, 176
Suicide, observations on, 19
———, remarkable cases of, 28, 470, 493
———, how far it is to be considered a criminal act, 29
———, utility of medical treatment, 25—470
———, the predisposition to commit, sometimes hereditary, 493
Switzerland, Dr. Forbes's tour in, 54
Symonds', Dr. J. A., Memoir of Dr. Prichard, 424
Tædium Vitæ, Brierre de Boismont on, 540
Tartar emetic, authorities in favour of its administration, 176
Temperaments, their influence on the human mind, 247
Tenon, his services in founding asylums in France, 430
Tobacco, debate on its use by the insane at Ohio Lunatic Asylum, 487
Treatment, medical, of insanity, by the ancients and moderns, 164
———, information upon, required by the commissioners, 179
———, necessity of early, 81, 175, 341
Treatment, moral, successful, 78
Types of insanity hereditary, 493
Verdicts of felo-de-se, 25
Verga, Dr., on the feeding of the insane, 219
Webster, Dr., on the bad effects of chloroform administered during parturition, 269
———, notes of a visit to the hospitals for the insane in France, 529
Wigan, Dr., on the duality of the mind, 11
Willis, Dr., his precautions in the case of George III., 177
Winslow, Dr. Forbes, on the plea of insanity in criminal cases, 448
Worship, divine, in lunatic asylums, 468

Savill & Edwards, Printers, 4, Chandos Street, Covent Garden.

